# Insulin Resistance and Platelet Hyperactivity: Hematological Insights and Nutritional Strategies for Vascular Protection

**DOI:** 10.3390/nu18050763

**Published:** 2026-02-26

**Authors:** Kiana Mohammadian, Narges Basirian, Fatemeh Fakhar, Shayan Keramat, Agata Stanek

**Affiliations:** 1 Division of Hematology and Blood Banking, Department of Medical Laboratory Sciences, School of Paramedical Sciences, Shiraz University of Medical Sciences, Shiraz 71348, Iran; kiana.mohammadian78@gmail.com (K.M.); fatemehfakhar1999@gmail.com (F.F.); 2 MSc in Hematology, Department of Hematology and Blood Transfusion, School of Paramedical Sciences, Bushehr University of Medical Sciences (BPUMS), Bushehr 7518759577, Iran; basirian1997@gmail.com; 3 VAS-European Independent Foundation in Angiology/Vascular Medicine, Via GB Grassi 74, 20157 Milan, Italy; shayan.sk1993@gmail.com; 4 Support Association of Patients of Buerger’s Disease, Buerger’s Disease NGO, Mashhad 9183785195, Iran; 5 Department of Internal Medicine, Metabolic Diseases and Angiology, Faculty of Health Sciences in Katowice, Medical University of Silesia, Ziołowa 45/47, 40-635 Katowice, Poland

**Keywords:** insulin resistance, platelet hyperactivity, nutritional strategies, thrombotic risk

## Abstract

Insulin resistance (IR) promotes a prothrombotic milieu by enhancing platelet hyperactivity, oxidative stress, and endothelial dysfunction, driving both microvascular and macrovascular complications in type 2 diabetes. Our review synthesizes mechanistic evidence showing that insulin-resistant platelets exhibit increased basal activation, elevated sensitivity to agonists, and reduced responsiveness to inhibitory signals, with distinct pro-aggregatory subpopulations amplifying thrombotic risk. Molecular pathways underlying platelet hyperactivation include reactive oxygen species accumulation, advanced glycation end-product signaling, disrupted calcium homeostasis, and impaired nitric oxide/prostacyclin pathways. Clinically, these mechanisms contribute to heightened arterial thrombosis, coronary artery disease, stroke, and microvascular injury, including nephropathy and retinopathy. Nutritional interventions emerge as effective modulators of platelet function and vascular health. Diets such as the Mediterranean, DASH, low-glycemic-index, and plant-based regimens, alongside bioactive compounds—including omega-3 fatty acids, polyphenols, vitamins D, E, C, and minerals like magnesium and zinc—may reduce platelet aggregation, oxidative stress, and systemic inflammation while restoring endothelial function. Clinical and epidemiological evidence demonstrates improvements in flow-mediated dilation, arterial elasticity, and stabilization of atherosclerotic plaques following dietary interventions. Integrating whole-diet strategies with targeted nutrients provides synergistic benefits, suggesting that personalized nutritional approaches can mitigate IR-induced platelet hyperactivity and lower vascular risk. These findings highlight nutrition as a practical, evidence-based adjunct to pharmacotherapy for cardiovascular protection in insulin-resistant populations.

## 1. Introduction

Insulin resistance (IR) and type 2 diabetes mellitus (T2DM) significantly elevate cardiometabolic risk, leading to various vascular complications, from microvascular damage such as nephropathy and retinopathy to macrovascular diseases such as coronary artery disease and stroke [1]. Increasing evidence emphasizes platelet hyperactivity as a key mechanism connecting metabolic imbalance to thrombotic incidents and vascular consequences [2,3].

Under physiological conditions, insulin regulates platelet activity, preventing excessive aggregation and preserving vascular homeostasis [4,5]. In insulin-resistant states, this regulatory effect is compromised, resulting in enhanced platelet susceptibility to circulating stimuli and a prothrombotic phenotype [6]. Metabolic stress, altered adipokine signaling, and intracellular dysregulation interact to drive platelet hyperactivation, increasing thrombotic and vascular risk in individuals with IR and T2DM [7]. Understanding these processes is essential for identifying potential strategies to modulate platelet function and preserve vascular health.

Among nutritional approaches, diet quality and specific dietary patterns play a pivotal role in mitigating cardiometabolic risk [8,9]. Diets such as the Mediterranean diet (MedDiet), low-glycemic-index (low-GI) diets, and the DASH diet have been associated with improved metabolic profiles, reduced inflammation, and beneficial effects on endothelial and platelet function [10]. Key bioactive constituents, including omega-3 fatty acids, polyphenols, carotenoids, vitamins, and minerals, exhibit antioxidant, anti-inflammatory, and insulin-sensitizing properties, potentially mitigating platelet hyperactivity and vascular dysfunction [11,12,13]. Epidemiological and clinical studies further support the association between adherence to these dietary strategies and reduced platelet aggregation, oxidative stress, and inflammation, reinforcing their cardioprotective potential [14,15].

Based on the current evidence, nutritional strategies represent a promising and practical approach to mitigate platelet hyperactivity and improve vascular outcomes in insulin-resistant and diabetic populations. This overview therefore provides the foundation for a detailed discussion of underlying pathophysiological mechanisms and dietary interventions.

**Figure 1:**
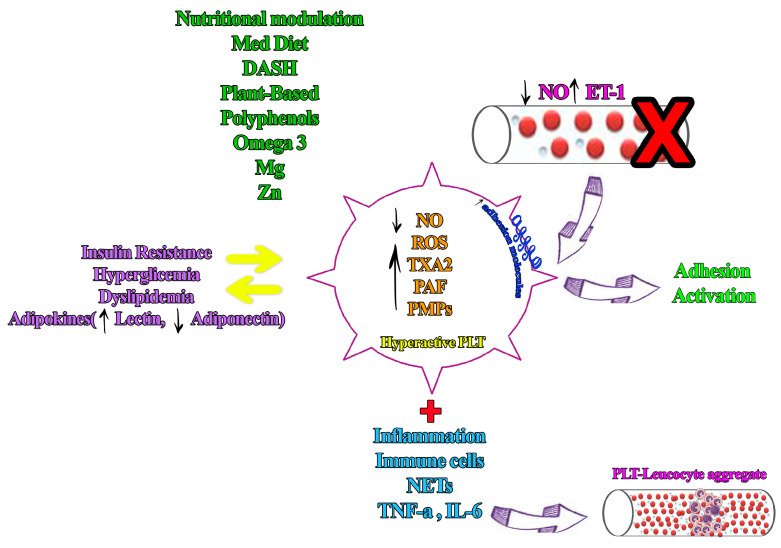
Vicious cycle involving hyperactive platelets, platelet–leukocyte aggregates, and endothelial dysfunction. Hyperactive platelets promote platelet–leukocyte aggregate formation and adhesion to the dysfunctional endothelium, enhancing leukocyte recruitment and vascular inflammation. Endothelial dysfunction (↓ NO, ↑ adhesion molecules) in turn exacerbates platelet hyperreactivity, continuing a cycle of thromboinflammation and endothelial injury. Nutritional interventions reduce platelet hyperactivity, inhibit aggregation, and break the vicious cycle.

Figure 1 schematically illustrates a platelet-centric vicious cycle linking insulin resistance, platelet hyperactivity, platelet–leukocyte interactions, and endothelial dysfunction, and highlights how dietary interventions may modulate platelet activation and disrupt thrombo-inflammatory vascular injury.

## 2. Pathophysiology

### 2.1. Insulin Receptors and Platelet Regulation

Human platelets express functional insulin receptors at densities comparable to classical insulin-responsive cells [16]. Upon insulin binding, multiple intracellular signaling cascades are activated, including IRS-1 phosphorylation and Gi protein modulation, which collectively maintain platelet quiescence and vascular homeostasis. Insulin increases cyclic nucleotides (cAMP and cGMP), reduces intraplatelet calcium, and enhances nitric oxide (NO) and prostacyclin (PGI2) signaling [17]. These pathways suppress platelet aggregation in response to diverse agonists such as ADP, thrombin, collagen, and platelet-activating factor (PAF), while also inhibiting thromboxane A2 (TXA2) synthesis and tissue factor activity, thereby supporting an anti-thrombotic platelet phenotype. These regulatory effects of insulin are crucial not only in maintaining platelet quiescence under normal conditions but also in preventing the early onset of platelet-mediated thrombotic events in insulin-sensitive individuals [1].

### 2.2. Platelet Hyperactivity in IR

In IR states such as T2DM and metabolic syndrome, insulin-mediated inhibition of platelet function is compromised. Platelets display basal hyperactivity, heightened sensitivity to circulating agonists, and diminished responsiveness to inhibitory signals like PGI2. Dysregulated adipokines, including leptin, resistin, PAI-1, and retinol binding protein 4 (RBP4), further impair insulin signaling in platelets, reinforcing a prothrombotic phenotype [18,19]. In type 1 diabetes, IR similarly exacerbates basal platelet activation, even before clinical vascular complications emerge, highlighting the early impact of impaired insulin signaling on platelet function [20]. Clinically, this manifests as a predisposition to both microvascular complications, such as nephropathy and retinopathy, and macrovascular events, including coronary artery disease and stroke. Elevated levels of inflammatory and oxidative mediators, such as ET-1, TNF-α, IL-6, and CRP, further sensitize platelets to activation in insulin-resistant states, compounding the prothrombotic milieu [21].

### 2.3. Molecular Mechanisms of Platelet Hyperactivation

Multiple molecular pathways contribute to platelet hyperactivation in IR. Chronic hyperglycemia increases reactive oxygen species (ROS) through aldose reductase activity, depleting NADPH, reducing glutathione levels, and accumulating advanced glycation end-products. ROS act as second messengers in thrombin- or collagen-stimulated platelets, altering intracellular Ca^2+^ flux and activating signaling cascades such as p38 MAPK and cytosolic phospholipase A2, ultimately enhancing thromboxane A2 synthesis and platelet aggregation [22]. Hyperglycemia also impairs mitochondrial function, causing membrane depolarization, cytochrome c release, and caspase activation, which generate platelet-derived microparticles (PMPs) that provide a prothrombotic surface [23]. Dyslipidemia, particularly oxidized LDL, exacerbates platelet activation via CD36-mediated MAPK and arachidonic acid pathways [24]. Disrupted calcium homeostasis, due to impaired Ca^2+^ ATPase and Na^+^/Ca^2+^ exchanger, increases cytosolic Ca^2+^ and activates calpains, remodeling the platelet proteome, enhancing α-granule secretion, and modifying adhesion molecule expression [25,26]. Endothelial dysfunction reduces NO and PGI2 bioavailability, increases ET-1, TNF-α, IL-6, and CRP levels, and fosters a prothrombotic endothelial environment. These molecular perturbations collectively prime platelets for exaggerated responses to circulating agonists, linking metabolic derangements directly to thrombotic risk [26,27].

### 2.4. Hematologic and Hemostatic Implications of Platelet Subpopulations

Hematologic studies demonstrate significant heterogeneity among platelet subpopulations in insulin-resistant individuals. Subsets expressing activation markers such as CD62P, PAC-1, and phosphatidylserine (PS) display pro-aggregatory and pro-coagulant phenotypes [28]. In insulin-resistant states, the proportion of fully activated platelets (CD62P^+^PAC-1^+^PS^+^) increases, while sensitivity to inhibitors such as PGI2 decreases, a phenomenon referred to as “priming.” These primed platelets have lower thresholds for activation and facilitate interactions with neutrophils and fibrinogen, promoting thrombus formation [20]. Understanding these subpopulations is critical for individualized antiplatelet strategies, as patients with a higher proportion of pro-aggregatory subsets may benefit from targeted therapies, especially when PGI2-mediated inhibition is impaired [29]. Notably, flow cytometry analyses reveal that IR amplifies basal platelet activation and reduces inhibitory responsiveness, reinforcing the need for precision medicine approaches in diabetic populations. Recognizing these functionally distinct platelet subpopulations enables identification of patients at higher thrombotic risk, potentially guiding individualized antiplatelet therapy and monitoring the efficacy of interventions targeting PGI2-insensitive platelets [24].

## 3. Clinical Consequences

### 3.1. Thrombosis in Diabetes Mellitus

Diabetes mellitus creates a profoundly prothrombotic milieu through a complex interplay of platelet hyperactivity, coagulation abnormalities, impaired fibrinolysis, and endothelial dysfunction [30,31]. Among these processes, platelet hyperactivity represents a central mechanistic driver linking metabolic dysregulation to thrombus formation. The heightened responsiveness of platelets is increasingly recognized as a central mechanism linking hyperglycemia and IR to thrombotic risk. Elevated urinary levels of thromboxane B_2_ and increased expression of platelet surface markers such as P-selectin and CD40L indicate a state of persistent platelet activation in both type 1 and type 2 diabetes [32]. At the molecular level, downregulation of prostacyclin receptors, upregulation of the ADP receptor P2Y_12_ and IGF1R, and glycation-driven alterations in membrane proteins amplify platelet aggregation and adhesion, thereby promoting intravascular thrombus formation. Advanced glycation end-products (AGEs) further activate platelets through RAGE and CD36 signaling, while oxidative stress pathways reinforce this hyperactive state and sustain platelet-driven thrombosis [33].

Beyond platelets, diabetes disrupts the delicate equilibrium between coagulation and fibrinolysis. Increased circulating levels of fibrinogen, prothrombin, and factors VII, VIII, IX, XI, and XII contribute to hypercoagulability, whereas concentrations of natural anticoagulants such as antithrombin, protein C, and protein S are reduced [32,34]. Concomitantly, fibrinolysis is suppressed by elevated levels of plasminogen activator inhibitor-1 (PAI-1), thrombin activatable fibrinolysis inhibitor (TAFI), and α2-antiplasmin, resulting in denser, fibrinolysis-resistant clots. In this setting, platelet-rich thrombi become more stable and resistant to endogenous fibrinolytic mechanisms. Hyperglycemia also promotes post-translational modifications of fibrinogen and plasminogen, further compromising clot resolution [35,36].

Endothelial dysfunction represents another cornerstone of the prothrombotic state. Chronic hyperglycemia and IR increase the burden of oxidative stress and reduce nitric oxide bioavailability, leading to impaired vasodilation, vascular stiffening, and enhanced platelet adhesion to the dysfunctional endothelium. Upregulation of adhesion molecules (CD31, CD36, CD62P, CD63) on both platelets and endothelial cells fosters cross-talk between thrombosis and inflammation, facilitating platelet adhesion, aggregation, and thrombus propagation at the vascular wall, thereby accelerating atherothrombosis [32,37]. Novel mechanisms have also emerged: platelets facilitate neutrophil extracellular trap (NET) formation, which further stabilizes platelet-rich thrombi and promotes vascular occlusion, while O-GlcNAcylation of platelet proteins under hyperglycemic conditions may further destabilize hemostatic balance [32,38].

Clinically, these mechanisms converge to explain the high incidence of arterial thrombosis in diabetes, particularly coronary artery disease, myocardial infarction, and ischemic stroke. Importantly, the role of platelets extends beyond passive thrombus formation to active participation in endothelial injury, plaque progression, and thrombotic complication development. Thus, from a hematologic standpoint, platelets function as key mediators of diabetic vascular complications, and their hyperactivity, coupled with impaired fibrinolysis, defines the unique platelet-driven thrombotic risk profile of patients with diabetes [37,39].

### 3.2. Vascular Complications of Diabetes

#### 3.2.1. Microvascular Complications of Diabetes

A large proportion of the morbidity and mortality associated with diabetes mellitus stems from its vascular complications, which encompass microvascular diseases such as retinopathy and nephropathy. Despite advances in glycemic control reducing the prevalence of microvascular lesions, cardiovascular disease remains the leading cause of death in T2DM, highlighting the complexity of diabetic vasculopathy beyond hyperglycemia alone [40].

The concept of diabetic panvascular disease (DPD) has been introduced to capture the systemic and diffuse nature of vascular injury in diabetes, whereby small vessels—from capillaries to arterioles—undergo pathological remodeling. This pathology includes endothelial dysfunction, basement membrane thickening, and microthrombosis. Importantly, these alterations create a permissive environment for platelet adhesion and microthrombus formation in the microcirculation. Importantly, microangiopathy predominantly manifests in target organs like the kidney and retina [41].

At the molecular level, endothelial dysfunction is considered the earliest and most central abnormality driving microvascular complications. IR and hyperglycemia reduce nitric oxide (NO) bioavailability, increase ROS, and upregulate vasoconstrictors such as endothelin-1. These alterations impair vasodilation, promote vascular stiffness, and facilitate leukocyte adhesion while simultaneously removing inhibitory constraints on platelet activation and aggregation within the microvasculature [42,43]. Simultaneously, oxidative stress and inflammatory signaling pathways—including protein kinase C (PKC), NF-κB, and JNK—promote vascular inflammation, increased permeability, and abnormal angiogenesis, thereby enhancing platelet–endothelium interactions and microvascular thrombus formation [44].

Beyond endothelial dysfunction, additional molecular mechanisms exacerbate microvascular damage. Hyperglycemia activates the polyol and hexosamine pathways, drives formation of advanced glycation end-products (AGEs), and induces mitochondrial dysfunction. AGEs engage RAGE receptors on endothelial and immune cells, amplifying NF-κB signaling, upregulating adhesion molecules, and enhancing inflammation, which further promotes platelet adhesion and microthrombi formation [45]. Dyslipidemia and free fatty acids stimulate systemic low-grade inflammation, macrophage M1 polarization, cytokine release (TNF-α, IL-6), and neutrophil extracellular trap (NET) formation. These processes stabilize platelet-rich microthrombi and reinforce a prothrombotic and hypofibrinolytic milieu within small vessels [46,47].

Clinically, microvascular complications such as diabetic retinopathy and nephropathy arise from endothelial apoptosis, pericyte and podocyte loss, microthrombosis, and abnormal angiogenesis [48]. Retinopathy remains the leading cause of blindness, while nephropathy is the primary cause of end-stage renal disease. From a hematologic perspective, platelets act as critical mediators, contributing to microthrombi formation and vascular injury [42,44]. Importantly, platelet activation contributes to microvascular injury through enhanced adhesion to damaged endothelium, release of pro-inflammatory mediators, and formation of platelet-derived microparticles, which amplify capillary occlusion, tissue ischemia, and progressive organ dysfunction [43,44].

#### 3.2.2. Macrovascular Complications of Diabetes

Macrovascular complications in diabetes, including coronary artery disease, myocardial infarction, and stroke, arise from a complex interplay of IR, chronic hyperglycemia, dyslipidemia, oxidative stress, and inflammation [49,50]. These processes converge to promote platelet activation and thrombus formation within large arteries. IR often develops years before overt hyperglycemia and contributes to endothelial dysfunction by impairing PI3K/Akt signaling, reducing GLUT-4-mediated glucose uptake, and decreasing endothelial nitric oxide (NO) production, thereby removing an important inhibitory restraint on platelet aggregation. Obesity exacerbates these effects by releasing free fatty acids (FFAs) and pro-inflammatory mediators, which activate Toll-like receptors and NF-κB, promoting vascular inflammation, leukocyte adhesion, and atherosclerotic plaque formation, while simultaneously enhancing platelet recruitment to sites of endothelial injury [51,52].

In parallel, hyperglycemia amplifies ROS generation via mitochondrial overload, PKC activation, polyol flux, and formation of advanced glycation end-products (AGEs). ROS directly react with NO to form peroxynitrite, further impairing endothelial function, thereby favoring platelet adhesion and activation, while PKC activation increases vasoconstrictors such as endothelin-1 (ET-1), enhances thromboxane A_2_ production, and reduces prostacyclin, shifting the vascular environment toward platelet-driven vasoconstriction and thrombosis. AGEs, through interaction with RAGE, stimulate inflammatory signaling, oxidative stress, and endothelial apoptosis, further promoting atherosclerosis and increasing the susceptibility to platelet-rich thrombus formation [52].

Dyslipidemia, particularly elevated saturated fatty acids such as palmitate, contributes to endothelial dysfunction and cell death via NOX4-dependent ROS production, NF-κB activation, and accumulation of lipid metabolites such as ceramides, which impair NO bioavailability, thereby facilitating platelet adhesion and aggregation [53,54]. Amino acid metabolism, including branched-chain amino acids, modulates macrophage polarization and atherosclerotic plaque formation, indirectly influencing platelet activation within the atherosclerotic milieu [55]. Endothelial heterogeneity further influences macrovascular outcomes: chronic hyperglycemia and IR impair angiogenesis, promote endothelial-to-mesenchymal transition (EndMT), and reduce regenerative capacity, which destabilizes the endothelial surface and favors platelet–endothelium interactions [56]. Dysregulation of VEGF/VEGFR2, NOTCH–Dll4, Kruppel-like factors, and epigenetic regulators such as LEENE lncRNA exacerbates vascular dysfunction, while endothelial microparticles released during apoptosis serve as early markers of vascular injury, and may further amplify platelet activation [57].

Together, these interconnected mechanisms—IR, hyperglycemia-induced ROS, PKC and AGE–RAGE signaling, dyslipidemia, and EndMT—create a pro-inflammatory, pro-oxidant, and prothrombotic milieu that drives macrovascular complications in diabetes. This integrated pathophysiology underlies accelerated atherosclerosis, impaired endothelial repair, and increased susceptibility to cardiovascular events, largely through sustained platelet activation, plaque-associated thrombosis, and thrombus propagation, emphasizing the need for therapeutic strategies that target metabolic, oxidative, and inflammatory pathways to attenuate platelet-driven macrovascular risk in diabetic patients [43,58]. These oxidative and inflammatory pathways not only accelerate atherosclerotic plaque formation but also directly increase platelet reactivity, promoting plaque-associated thrombosis and acute coronary events [51].

Taken together, both microvascular and macrovascular diabetic complications share a common hematologic denominator: platelet hyperactivity. Platelets act not only as passive responders to endothelial injury but also as active inflammatory and thrombotic amplifiers, driving platelet adhesion, aggregation, and thrombus formation within diseased vessels, thereby representing a mechanistic bridge between insulin resistance and vascular disease [54].

## 4. Nutritional Strategies

### 4.1. Dietary Patterns Beyond MedDiet

Dietary patterns extending beyond the traditional MedDiet—including the DASH diet, plant-based diets, low-glycemic-index (low-GI) diets, low-carbohydrate diets, and calorie restriction (CR)—have demonstrated beneficial effects on metabolic control, cardiovascular risk factors, and vascular function [15]. These effects are increasingly recognized to involve modulation of platelet activation and thrombotic risk. Clinical trials in adults with type 2 diabetes have shown that higher adherence to the DASH diet for eight weeks significantly reduced body weight, blood pressure, and markers of inflammation, including CRP and CXCL4 [59], a chemokine closely linked to platelet activation. The DASH diet emphasizes fruits, vegetables, whole grains, low-fat dairy, legumes, nuts, and seeds while reducing sodium, saturated fats, and sugar intake. Its effects on metabolic health may be mediated by improvements in insulin sensitivity, reductions in systemic inflammation, and modulation of endothelial function, thereby indirectly attenuating platelet activation [60,61]. Mechanistically, the DASH diet reduces serum levels of TLR-4, MCP-1, and LPS, lowers oxidative stress, and has been associated with reduced platelet activation. Observational studies also link DASH adherence to lower IL-6, CRP, and TNF-α levels, highlighting its anti-inflammatory and antioxidant properties, which contribute to a less prothrombotic platelet phenotype [62,63].

Calorie restriction, defined as a 20–30% reduction in daily caloric intake while maintaining nutrient quality, improves total and visceral adiposity, reduces ectopic fat deposition, and lowers blood pressure [64]. Mechanistic studies show that CR decreases insulin resistance, oxidative stress, and inflammatory biomarkers, including F2-isoprostanes and platelet-derived growth factor AB, factors known to influence platelet reactivity. CR-induced weight loss also enhances flow-mediated dilation and arterial elasticity, thereby reducing platelet adhesion to the vascular wall and thrombotic susceptibility, contributing to cardiovascular protection [65].

Plant-based diets, including vegetarian or flexitarian approaches, focus on whole plant foods and minimize red and processed meat intake. Evidence demonstrates that these diets improve metabolic syndrome components, reduce obesity risk, lower incidence of diabetes and cardiovascular disease, and favorably modulate inflammatory and oxidative stress markers. These vascular and metabolic benefits are accompanied by reduced platelet activation and aggregation, partly attributable to bioactive compounds such as polyphenols, flavonoids, and antioxidant vitamins (C and E) [65,66].

Dietary nitrate from vegetables such as spinach enhances nitric oxide bioavailability, reduces blood pressure, inhibits platelet aggregation, and improves endothelial function [67,68]. Other compounds such as lycopene, catechins, quercetin, hydroxytyrosol (from olive oil), curcuminoids, ginsenosides, and aged garlic extract exert antioxidant, anti-inflammatory, antiplatelet, and vascular-protective effects, thereby modulating platelet-driven thrombotic pathways [69].

The MedDiet remains a robust model for cardiovascular and metabolic health. It emphasizes olive oil, nuts, fruits, vegetables, whole grains, fish, and moderate wine consumption while limiting red meat and sugar. Its benefits include improved insulin sensitivity, favorable lipid profiles, reduced oxidative stress, enhanced endothelial function, and lower incidence of cardiovascular disease and T2DM [70]. Importantly, these effects translate into reduced platelet activation and aggregation. Functional components such as oleic acid, polyphenols, and carotenoids contribute to anti-inflammatory, antioxidant, and antiplatelet actions [71].

Overall, dietary patterns beyond the MedDiet—particularly DASH, calorie restriction, and plant-based diets—exert multifaceted effects on metabolic and cardiovascular health through modulation of inflammation, oxidative stress, endothelial function, and platelet activity. Integrating these diets into lifestyle interventions offers a potent approach to prevent and manage metabolic syndrome, T2DM, hypertension, and associated cardiovascular complications, in part by attenuating platelet-driven thrombosis [66].

Recent intervention studies have highlighted that Mediterranean dietary patterns and other plant-based diets can modulate novel biomarkers of platelet function and inflammation, including platelet-activating factor (PAF) and lipoprotein-associated phospholipase A_2_ (Lp-PLA_2_). In four intervention studies examining the MedDiet, significant reductions in PAF-induced platelet aggregation were observed, particularly among individuals with type 2 diabetes, who exhibited higher baseline platelet hyperactivity [72]. Similarly, Lp-PLA_2_ activity was favorably altered in HDL following MedDiet interventions supplemented with extra-virgin olive oil, suggesting additional platelet-related mechanisms of cardiovascular protection. Conversely, Western dietary patterns were associated with higher levels of Lp-PLA_2_, reinforcing the role of dietary quality in modulating platelet-mediated prothrombotic and inflammatory pathways [73,74].

Beyond these dietary models, the quality of dietary fats plays a crucial mechanistic role in platelet modulation. High-saturated-fat diets have been associated with impaired platelet signaling, whereas polyunsaturated fatty acid (PUFA)-rich diets prevent these alterations [75]. Dietary fatty acids incorporate into megakaryocyte and platelet phospholipids, influencing membrane composition, receptor signaling, and platelet reactivity. PUFA availability also modulates megakaryocyte maturation and proplatelet formation through CD36-dependent fatty-acid transfer from adipocytes, linking dietary fat composition directly to platelet production and function. These insights provide additional justification for dietary patterns emphasizing unsaturated fats in insulin-resistant populations [72,76].

### 4.2. Nutritional Compounds and Bioactive Molecules

Specific dietary components such as omega-3 fatty acids, polyphenols, vitamins D, E, C, and minerals like magnesium and zinc play critical roles in modulating platelet hyperactivity and vascular outcomes [13,72]. As described in Section 3, platelet hyperactivation in insulin-resistant states is driven by oxidative stress, inflammatory signaling, and impaired inhibitory pathways. Omega-3 fatty acids have been shown to attenuate thromboxane-mediated platelet aggregation, and may modulate oxidative and inflammatory pathways involved in platelet activation, while supporting endothelial function [77,78]. Polyphenols such as resveratrol, hydroxytyrosol, quercetin, and epigallocatechin gallate exhibit antiplatelet, antioxidant, and anti-inflammatory effects, which have been associated with reduced platelet hyperactivity in hyperglycemic conditions [11,79]. Vitamin D modulates platelet activation, endothelial function, and systemic inflammation, whereas vitamins E and C have been associated with reduced oxidative stress, inhibition of platelet aggregation, and preservation of nitric oxide bioavailability. Magnesium and zinc act as natural platelet antagonists, reducing aggregation and thromboxane formation, with deficiencies frequently observed in diabetic populations, which may further exacerbate platelet hyperreactivity [13,80].

Nuts and legumes, abundant sources of polyphenols, flavonoids, phenolic acids, and essential nutrients, have emerged as modulators of platelet function through both direct biochemical effects and epigenetic regulation [81]. Compounds such as catechins, quercetin, genistein, and caffeic acid have been shown to influence platelet signaling pathways, including cyclooxygenase activity and thromboxane A_2_ production, thereby being associated with reduced platelet aggregation. Additionally, these bioactive nutrients contribute methyl donors and may modulate DNA methylation of genes involved in platelet activation, such as PEAR1, linking dietary intake to epigenetic regulation of platelet hyperactivity [82]. The combination of polyphenol-rich foods and nutrients supporting one-carbon metabolism (folate, vitamin B12, methionine, choline) enhances S-adenosylmethionine availability, which may stabilize methylation patterns relevant to platelet function and vascular homeostasis. These observations support a potential dual anti-inflammatory and antithrombotic role of regular nut and legume consumption, although causal relationships require further confirmation [81,83].

**Figure 2:**
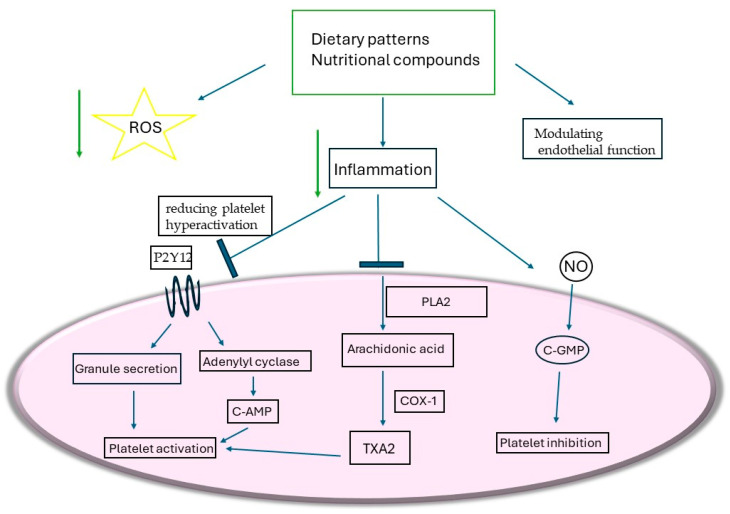
Consumption of nutritional compounds, bioactive molecules and diets like the Mediterranean, DASH and plant-based diets has useful outcomes comprising modulation of endothelial function, reducing inflammation and oxidative stress. This outcome leads to reducing platelet hyperactivation due to inhibition of TXA2 and P2Y12, and also modulation of the NO pathway.

Emerging evidence also implicates gut microbiota-derived metabolites in diet–platelet interactions. As outlined in Section 3, microbial metabolites have been linked to platelet hyperreactivity and thrombotic risk. High-fat and animal-protein-rich diets elevate circulating trimethylamine-N-oxide (TMAO), which has been associated with enhanced platelet responsiveness [84]. Conversely, fiber-rich and plant-forward dietary patterns reduce TMAO production through microbial modulation and increased short-chain fatty acid formation, potentially lowering platelet activation and vascular risk. These microbiota-mediated effects represent an additional, indirect axis through which nutrition may influence platelet phenotypes, although human interventional evidence remains limited [85] (Figure 2).

### 4.3. Diet-Induced Adipose Modulation and Downstream Effects on Platelet Activity

High-fat dietary patterns not only induce hypercholesterolemia and triglyceride elevation but also alter adipose tissue-derived signals that influence platelet activation. As described in Section 3, dyslipidemia and adipose tissue dysfunction create a pro-activatory environment for platelets in insulin-resistant states. Diets rich in saturated and trans fats have been associated with elevations in leptin and reductions in adiponectin, which may contribute to a prothrombotic milieu and enhanced platelet aggregation. Experimental findings indicate that high-fat-induced dyslipidemia is associated with increased circulating markers of platelet activation, including β-thromboglobulin, P-selectin, and platelet factor-4 [86,87]. Moreover, high-fat diets have been linked to elevated proprotein convertase subtilisin/kexin type 9 (PCSK9), which may potentiate platelet activation and exacerbate hyperlipidemia-related vascular risk. Taken together, these observations suggest that the quality of dietary fat may influence adipose–platelet signaling and platelet hyperreactivity, supporting dietary fat composition as a potentially modifiable target in nutritional strategies for insulin resistance and cardiometabolic disease [86].

### 4.4. Mechanistic Links to Platelet Hyperactivity

As outlined in Section 3, IR promotes platelet hyperactivity via multiple mechanisms, including increased oxidative stress, pro-inflammatory signaling, and impaired endothelial NO production. These mechanisms create a pro-activatory platelet environment in insulin-resistant states. Excess reactive oxygen species have been shown to enhance platelet activation and thromboxane A_2_ formation, while reducing prostacyclin- and NO-mediated inhibition of aggregation [12].

Obesity and high-fat diets have been associated with changes in the platelet transcriptome, lipidome, and signaling pathways, contributing to platelet hyperreactivity in vivo, despite occasionally reduced responsiveness in isolated platelets. Platelets from obese individuals exhibit alterations in signaling pathways, including Src family kinase activity and GPVI expression, as well as distinct lipidomic signatures characterized by reduced phosphatidylcholine and phosphatidylethanolamine [88]. These alterations are linked to enhanced thrombotic susceptibility and provide a metabolic context in which nutritional interventions may be particularly relevant. Recent evidence also highlights a bidirectional relationship between platelets and glucose homeostasis. Platelets release bioactive lipid mediators such as 20-HETE, which have been implicated in modulation of glucose-stimulated insulin secretion through FFAR1–PKD1 signaling. Dietary patterns that improve insulin sensitivity—such as low-glycemic, Mediterranean, and plant-based diets—may indirectly influence these platelet-derived lipid mediators, potentially reducing platelet hyperreactivity and metabolic strain on pancreatic β-cells. These interactions appear to be modified by aging and dietary fat exposure, underscoring the interplay between diet quality, metabolic status, and platelet biology [89].

Building on platelet signaling pathways described in Section 3, bioactive nutrients may modulate key platelet aggregation pathways, including P2Y_12_, TXA_2_, cAMP/cGMP, and NO signaling, and have been associated with suppression of pro-inflammatory mediators such as TNF-α and IL-6, thereby supporting endothelial function and attenuating platelet hyperactivation [11]. The integrated effects of dietary patterns and bioactive foods on platelet function, epigenetic regulation, and inflammatory biomarkers are summarized in Table 1. As summarized in Table 1, dietary interventions consistently target platelet hyperreactivity through multiple convergent mechanisms, including attenuation of oxidative stress, modulation of inflammatory signaling, and preservation of endothelial nitric oxide bioavailability. Importantly, these effects extend beyond single nutrients and highlight the relevance of whole-diet patterns in platelet-driven thrombotic risk.

Emerging evidence demonstrates that platelets actively participate in metabolic regulation through bidirectional interactions with adipose tissue. Adipokines such as leptin and adiponectin have been shown to influence platelet behavior, with leptin enhancing platelet aggregation and responsiveness to vascular injury, while adiponectin generally exerts antithrombotic effects [90]. Conversely, activated platelets release dense-granule mediators—including ADP, ATP, and serotonin—which may influence adipocyte metabolism, including lipolysis, insulin sensitivity, and adipokine secretion. Platelet-derived TGF-β and thrombospondin-1 have been implicated in impaired adipose tissue browning and metabolic dysfunction. Collectively, these observations support the existence of a platelet–adipocyte regulatory axis that may exacerbate insulin resistance and platelet hyperreactivity in obesity, highlighting a potential target for dietary and metabolic interventions [86].

## 5. Discussion

IR drives a prothrombotic milieu through platelet hyperactivity, oxidative stress, chronic inflammation, and endothelial dysfunction, thereby linking metabolic dysregulation directly to platelet-mediated thrombus formation in both microvascular and macrovascular complications [20]. Platelet subpopulations exhibit heterogeneous pro-aggregatory phenotypes in insulin-resistant states, amplifying platelet-driven thrombotic susceptibility and complicating vascular outcomes. Given the intricate interplay between metabolic and hematologic pathways, nutritional strategies have emerged as pivotal modulators of platelet function and vascular health [1,16,74].

Dietary patterns such as the MedDiet, DASH, low-glycemic-index, plant-based, and calorie restriction regimens collectively demonstrate the capacity to improve insulin sensitivity, reduce systemic inflammation, and enhance endothelial function [12,52,58,59]. Clinical evidence highlights that adherence to these diets lowers body weight, blood pressure, and circulating inflammatory markers such as CRP, IL-6, TNF-α, CXCL4, TLR-4, MCP-1, and LPS, thereby attenuating platelet activation and promoting platelet-dependent vascular homeostasis. These beneficial effects arise from synergistic interactions among bioactive compounds, fiber, unsaturated fatty acids, antioxidants, and micronutrients, rather than from isolated nutrients [77,81].

Specific nutritional constituents play mechanistic roles in modulating platelet hyperactivity. Omega-3 fatty acids inhibit thromboxane-mediated aggregation and oxidative stress, polyphenols and flavonoids reduce inflammatory signaling and oxidative damage, vitamins D, E, and C maintain nitric oxide bioavailability and platelet quiescence, and minerals such as magnesium and zinc act as natural platelet antagonists [83,84,85]. Collectively, these bioactives interact with intracellular platelet pathways—including P2Y_12_, TXA_2_, cAMP/cGMP, and NO signaling—and suppress pro-inflammatory mediators, thereby restoring endothelial function and limiting platelet-driven thrombotic potential. Functional foods rich in nitrates, lycopene, hydroxytyrosol, curcuminoids, and ginsenosides further complement these effects by enhancing nitric oxide bioavailability, reducing oxidative stress, and supporting antiplatelet vascular protection [86,87].

Integration of whole dietary patterns with these bioactive compounds translates into clinically meaningful platelet-related outcomes. Trials indicate reductions in ADP- and PAF-induced platelet aggregation, improvements in flow-mediated dilation and arterial elasticity, and stabilization of atherosclerotic plaque. The combination of whole-diet interventions with targeted nutrients provides synergistic benefits, emphasizing that single-nutrient supplementation may be insufficient to fully counteract platelet hyperactivity in insulin-resistant populations [88,89].

From a translational perspective, personalized nutritional strategies—tailored to insulin sensitivity, platelet subpopulation profiles, metabolic status, and cardiovascular risk—may optimize platelet-mediated vascular protection. Monitoring biomarkers of oxidative stress, inflammation, and platelet activity may inform dietary prescriptions and evaluate intervention efficacy. Furthermore, interactions among dietary fiber, polyphenols, healthy fats, and gut microbiota may represent additional mechanisms through which diet modulates platelet-dependent thrombotic risk [90,91,92]. Collectively, this review uniquely integrates insulin resistance, platelet biology, and nutritional strategies, highlighting platelets as mechanistic mediators and nutrition as a modifiable regulator of thrombotic risk—a perspective not systematically addressed in prior reviews.

## 6. Translational and Clinical Implications

From a clinical perspective, nutritional interventions may represent a feasible adjunct strategy to pharmacological antiplatelet therapy in insulin-resistant populations. Standard antiplatelet agents such as aspirin and P2Y12 inhibitors primarily target cyclooxygenase-1 and ADP-mediated signaling; however, insulin resistance is associated with broader platelet dysregulation driven by oxidative stress, endothelial dysfunction, chronic inflammation, and altered lipid metabolism [93,94]. Dietary patterns rich in fiber, polyphenols, and unsaturated fatty acids may modulate these upstream drivers, thereby complementing conventional antiplatelet drugs [95]. Importantly, nutrition-based interventions may be particularly relevant in individuals with high residual platelet reactivity, obesity-related inflammation, or aspirin resistance, where platelet activation persists despite pharmacotherapy. While direct evidence from large randomized trials is still limited, current mechanistic and clinical studies suggest that dietary optimization may enhance vascular protection by improving endothelial nitric oxide bioavailability, reducing oxidative stress, and attenuating platelet priming. Nevertheless, robust long-term randomized controlled trials are required to determine whether platelet-modulating dietary strategies translate into reduced clinical thrombotic events [95,96].

Personalized nutritional strategies tailored to individual insulin sensitivity, platelet subpopulation profiles, and cardiovascular risk may optimize vascular protection. Such interventions not only serve preventive roles—attenuating platelet hyperactivity and endothelial dysfunction in at-risk populations—but may also contribute to reversal or mitigation of established metabolic and vascular abnormalities. Monitoring biomarkers of oxidative stress, inflammation, and platelet activity can guide dietary prescriptions and evaluate intervention efficacy, allowing for precision nutrition approaches in insulin-resistant individuals [94,96].

## 7. Clinical Relevance of Platelet Heterogeneity and Subpopulations

Increasing evidence indicates that platelet populations are functionally heterogeneous, with distinct subpopulations exhibiting differential pro-aggregatory, pro-inflammatory, and pro-thrombotic phenotypes in insulin-resistant and diabetic states [31,44,97]. Expansion of hyperreactive platelet subpopulations may contribute to residual thrombotic risk despite standard antiplatelet therapy, helping to explain the persistently elevated cardiovascular risk observed in these patients [31,44].

From a diagnostic perspective, assessment of platelet activation markers, aggregation responses, and platelet-derived inflammatory mediators may improve vascular risk stratification beyond traditional metabolic biomarkers in insulin-resistant populations [32,42,44]. Such platelet phenotyping could help identify individuals with heightened platelet-driven thrombotic risk and suboptimal response to conventional therapy [37,39].

From a therapeutic and translational standpoint, recognition of platelet heterogeneity supports more individualized antithrombotic and lifestyle-based strategies. Patients characterized by increased platelet hyperreactivity may particularly benefit from combined pharmacological and nutritional interventions targeting upstream metabolic, inflammatory, and oxidative drivers of platelet activation [66,72,89]. In this context, dietary modulation of platelet function may represent a complementary approach to standard antiplatelet therapy and an integral component of emerging precision nutrition strategies in insulin-resistant populations [72,89].

## 8. Conclusions

IR induces a prothrombotic state through platelet hyperactivity, oxidative stress, inflammation, and endothelial dysfunction, thereby directly linking metabolic dysregulation to platelet-mediated thrombus formation and contributing to both micro- and macrovascular complications. Hematologic insights reveal functional heterogeneity among platelet subpopulations, underscoring the need for precision, platelet-targeted approaches to mitigate thrombotic risk. Nutritional strategies—particularly dietary patterns rich in fiber, unsaturated fatty acids, polyphenols, and essential micronutrients—may improve insulin sensitivity, reduce oxidative stress, and modulate platelet aggregation pathways. Evidence from clinical studies supports the integration of dietary interventions as adjuncts to pharmacological therapy, highlighting their potential to attenuate platelet hyperactivity and enhance vascular outcomes. Future research should prioritize personalized dietary approaches informed by platelet subpopulation profiles, the combined effects of multiple bioactive nutrients, and longitudinal evaluation of platelet dynamics in insulin-resistant populations. Collectively, these insights position nutrition as a pivotal, modifiable modulator of platelet-driven thrombotic risk, bridging mechanistic understanding with practical clinical application.

## Figures and Tables

**Table 1:** Impact of dietary patterns and bioactive foods on platelet function, epigenetic modulation, and inflammatory biomarkers.

Dietary Pattern/Food	Mechanisms/Active Compounds	Effect on Platelets (PA/Lp-PLA2)	Epigenetic/DNA Methylation Effects	Effect on Inflammation/Other Biomarkers	Evidence/Study Type
Mediterranean diet	Olive oil, nuts, fruits, vegetables, fish, polyphenols	↓ PAF-induced aggregation, ↓ Lp-PLA2 in HDL	-	↓ IL-6, CRP, TNF-α, improved endothelial function	RCTs and intervention studies
DASH diet	Fruits, vegetables, whole grains, low-fat dairy, nuts, seeds, reduced sodium and saturated fat	↓ PA, ↑ NO	-	↓ CRP, CXCL4, TNF-α	RCTs, adults with T2DM
Calorie restriction (CR)	20–30% caloric reduction, nutrient-dense diet	Indirect ↓ PA via weight loss and reduced visceral fat	-	↓ F2-isoprostanes, PDGF-AB, improved arterial elasticity	RCTs, mechanistic studies
Plant-based/Vegetarian diet	Vegetables, legumes, nuts, polyphenols, flavonoids, vitamins C and E, folate	↓ PA, ↓ Lp-PLA2	Provides methyl donors, enhances SAM, stabilizes DNA methylation of platelet-related genes	↓ LDL oxidation, reduced oxidative stress	RCTs, pre-post interventions
Nuts (walnuts, almonds, pistachios, hazelnuts)	Polyphenols (catechins, quercetin, phenolic acids), MUFA/PUFA, vitamin E	↓ PA, ↓ TXA2 production, ↓ PDMP release	DNMT inhibition, stabilizes PEAR1 methylation	↓ ROS, improved NO bioavailability	RCTs, in vitro, animal studies
Legumes (soy, chickpeas, beans)	Isoflavones (genistein, daidzein), flavonoids, folate	↓ PA, ↓ Lp-PLA2	Supports SAM production, methylation of PA-related genes	↓ oxidative stress, improved lipid profiles	RCTs, in vitro studies
Western diet	Processed foods, sugary beverages, processed meats	↑ Lp-PLA2, ↑ PA	-	↑ TNF-α, IL-6, CRP	Observational/cohort studies
Gut microbiota-mediated effects	Fiber, probiotics, polyphenols	↓ PA via ↓ TMAO production	-	↓ inflammation, ↓ oxidative stress	Mechanistic studies, in vivo
High-fat/Saturated fat diet	Saturated and trans fats	↑ PA, impaired platelet signaling	-	↑ leptin, ↓ adiponectin, ↑ PCSK9	Mechanistic studies, animal/in vitro

PA—Platelet Aggregation; Lp-PLA2—Lipoprotein-associated Phospholipase A2; NO—Nitric Oxide; TXA2—Thromboxane A2; PDMP—Platelet-Derived Microparticles; DNMT—DNA Methyltransferase; SAM—S-Adenosylmethionine; TMAO—Trimethylamine-N-oxide; CRP—C-Reactive Protein; CXCL4—Chemokine (C-X-C motif) Ligand 4; PDGF-AB—Platelet-Derived Growth Factor AB; MUFA/PUFA—Monounsaturated/Polyunsaturated Fatty Acids; T2DM—Type 2 Diabetes Mellitus.

## Data Availability

We used PubMed, SCOPUS, and ScienceDirect databases to screen articles for this review. We did not report any data.

## Abbreviations

AGE	Advanced Glycation End-product
CR	Calorie Restriction
CRP	C-Reactive Protein
DPD	Diabetic Panvascular Disease
EndMT	Endothelial-to-Mesenchymal Transition
ET-1	Endothelin-1
FFA	Free Fatty Acid
IR	Insulin Resistance
Lp-PLA2	Lipoprotein-associated Phospholipase A2
MedDiet	Mediterranean Diet
NET	Neutrophil Extracellular Trap
NO	Nitric Oxide
PAI-1	Plasminogen Activator Inhibitor-1
PCSK9	Proprotein Convertase Subtilisin/Kexin Type 9
PGI2	Prostacyclin
PKC	Protein Kinase C
PMP	Platelet-Derived Microparticle
PUFA	Polyunsaturated Fatty Acid
RBP4	Retinol Binding Protein 4
ROS	Reactive Oxygen Species
T2DM	Type 2 Diabetes Mellitus
TMAO	Trimethylamine-N-Oxide
TXA2	Thromboxane A2
